# An Improved Culture Method for Selective Isolation of *Campylobacter jejuni* from Wastewater

**DOI:** 10.3389/fmicb.2016.01345

**Published:** 2016-08-26

**Authors:** Jinyong Kim, Euna Oh, Graham S. Banting, Shannon Braithwaite, Linda Chui, Nicholas J. Ashbolt, Norman F. Neumann, Byeonghwa Jeon

**Affiliations:** ^1^School of Public Health, University of Alberta, Edmonton, ABCanada; ^2^Provincial Laboratory for Public Health, Alberta Health Services, Edmonton, ABCanada; ^3^Department of Laboratory Medicine and Pathology, University of Alberta, Edmonton, ABCanada

**Keywords:** *Campylobacter jejuni*, wastewater, isolation, antibiotics, qRT-PCR

## Abstract

*Campylobacter jejuni* is one of the leading foodborne pathogens worldwide. *C. jejuni* is isolated from a wide range of foods, domestic animals, wildlife, and environmental sources. The currently available culture-based isolation methods are not highly effective for wastewater samples due to the low number of *C. jejuni* in the midst of competing bacteria. To detect and isolate *C. jejuni* from wastewater samples, in this study, we evaluated a few different enrichment conditions using five different antibiotics (i.e., cefoperazone, vancomycin, trimethoprim, polymyxin B, and rifampicin), to which *C. jejuni* is intrinsically resistant. The selectivity of each enrichment condition was measured with *C*_t_ value using quantitative real-time PCR, and multiplex PCR to determine *Campylobacter* species. In addition, the efficacy of *Campylobacter* isolation on different culture media after selective enrichment was examined by growing on Bolton and Preston agar plates. The addition of polymyxin B, rifampicin, or both to the Bolton selective supplements enhanced the selective isolation of *C. jejuni*. The results of 16S rDNA sequencing also revealed that *Enterococcus* spp. and *Pseudomonas aeruginosa* are major competing bacteria in the enrichment conditions. Although it is known to be difficult to isolate *Campylobacter* from samples with heavy contamination, this study well exhibited that the manipulation of antibiotic selective pressure improves the isolation efficiency of fastidious *Campylobacter* from wastewater.

## Introduction

*Campylobacter* is the major bacterial cause of foodborne infection, annually accounting for approximately 166 million foodborne illnesses around the world (Kirk et al., 2015). In addition to the clinical symptoms of gastroenteritis, *Campylobacter* is the major risk factor of Guillain–Barré syndrome (GBS), a neurological disorder causing muscular paralysis, as a post-infection complication (Hughes and Cornblath, 2005). Among pathogenic *Campylobacter* species, *C. jejuni* and *C. coli* are most frequently associated with human infection (Kaakoush et al., 2015). Thus far, the consumption of contaminated poultry is the primary cause of developing human campylobacteriosis (Whiley et al., 2013).

Despite the well-known fastidious nature of *Campylobacter* (Silva et al., 2011), *Campylobacter* is isolated from environmental sources, such as lake, river, sea, and sewage, suggesting that environmental water is a possible vehicle that transmits *Campylobacter* to humans (Jones, 2001). *C. jejuni* is the pathogenic species that is mainly related to water-borne campylobacteriosis worldwide (Pitkanen, 2013). In Canada, *Campylobacter* outbreaks caused by cross contamination related with meltwater and heavy rainfall are problematic to public health (Millson et al., 1991; Clark et al., 2003). However, the isolation of *Campylobacter* implicated in water-borne outbreak appear to be challenging, not only due to rapid loss in culturability of isolates from the environment (Wingender and Flemming, 2011) and from stool samples (Bullman et al., 2012), but also due to the time gap between the initial infection and outbreak investigation (Hanninen et al., 2003; Jakopanec et al., 2008). Therefore, regular monitoring system of water resources by using culture-based methods is likely to underestimate the prevalence of *Campylobacter* spp. in the environment. This might mislead our understanding of the role played by the environmental sources in human infection and possibly the contamination of food chain by *Campylobacter*, even though *Campylobacter* is most frequently detected in animal fecal samples (29.7%), untreated human sewage (25.6%), and surface water (26.6%), according to a study in Alberta, Canada, among the three major foodborne pathogens, including *Campylobacter*, *Salmonella*, and *Escherichia coli* O157:H7 (Jokinen et al., 2011).

Various culture supplements have been examined to improve selective isolation of *Campylobacter* spp. (Corry et al., 1995). For example, ISO method 2005 has been applied for the detection of thermo-tolerant campylobacters from water, and alternative culture-based methods in combination with molecular end-point confirmation (Hokajarvi et al., 2013; Pitkanen, 2013). Sample volume, incubation time, enrichment volume, passage of enrichment, and PCR-primer specificity all play an important role (Levesque et al., 2011) and enrichment procedures as well (Rosef et al., 2001). Khan et al. (2009, 2013) compared two methods (i.e., centrifugation vs. membrane filtration) for the isolation and detection of *Campylobacter* from agriculture watersheds, and reported the effect of incubation temperature on the detection rates and the type of dominant *Campylobacter* species detected from water samples. However, wastewater samples are even more challenging than samples from agricultural watersheds due to the relatively low number of *Campylobacter* in comparison with the high levels of microbial competitors and PCR inhibitors in wastewater (Koenraad et al., 1997; Abulreesh et al., 2005; Schrader et al., 2012).

To overcome the limitations in traditional culture-based methods for the detection of foodborne pathogens, molecular methods, such as PCR, have become practical and widely used due to the speed and reproducibility (Law et al., 2015). Recently, direct quantitative PCR was applied to the detection of *Campylobacter* in river water and showed the possibility of an alternative method for *Campylobacter* detection (Van Dyke et al., 2010). Nevertheless, the detection validation of *Campylobacter* with culture-based methods would still be necessary to reveal the direct correlation between a clinical illness and its etiological agents.

In this study, we present amendments to existing culture methods to improve the enrichment and isolation of *Campylobacter* spp. from wastewater. We used raw sewage influent samples since they are contaminated more heavily than effluent samples. By adopting different incubation temperatures and several antibiotics, to which *C. jejuni* is intrinsically resistant, we developed an improved enrichment method to recover culturable *C. jejuni* from wastewater samples. The efficiency of *Campylobacter* isolation was evaluated using quantitative real-time PCR (qRT-PCR) targeting genus-specific 16S rDNA primers, and a second end-point multiplexed PCR with species-specific primers. By using 16S rDNA amplicon sequencing, in addition, we identified the major bacteria in wastewater that compete with *Campylobacter* under the selective enrichment conditions.

## Materials and Methods

### Bacterial Strains, Culture Conditions, and Primers

*Campylobacter jejuni* ATCC 33560 and NCTC 11168 were routinely cultured in Mueller Hinton (MH) media at 42°C under microaerobic conditions (5% O_2_, 10% CO_2_, and 85% N_2_). The primers used in the study are described in **Table 1**.

**Table 1:** Primers used in this study.

Primer	Sequence (5′→ 3′)	Reference
*CampyLvl-16S-F	CCT GAM GCA GCA ACG CC	de Boer et al., 2013
*CampyLvl-16S-R	CGG AGT TAG CCG GTG CTT ATT	
*CampyLvl-16S-P	CTC CGA AAA GTG TCA TCC T	
**CampyYM-16S-F	GGA TGA CAC TTT TCG GAG C	Yamazaki-Matsune et al., 2007
**CampyYM-16S-R	CAT TGT AGC ACG TGT GTC	
**C. hyointest-23S-F	ATA ATC TAG GTG AGA ATC CTA G	
**C. hyointest-23S-R	GCT TCG CAT AGC TAA CAT	
**C. coli-ask-F	GGT ATG ATT TCT ACA AAG CGA G	
**C. coli-ask-R	ATA AAA GAC TAT CGT CGC GTG	
**C. fetus-cstA-F	GGT AGC CGC AGC TGC TAA GAT	
**C. fetus-cstA-R	AGC CAG TAA CGC ATA TTA TAG TAG	
**C. lari-glyA-F	TAG AGA GAT AGC AAA AGA GA	
**C. lari-glyA-R	TAC ACA TAA TAA TCC CAC CC	
**C. jejuni-cj0414-F	CAA ATA AAG TTA GAG GTA GAA TGT	
**C. jejuni-cj0414-R	CCA TAA GCA CTA GCT AGC TGA T	
**C. upsal-lpxA-F	CGA TGA TGT GCA AAT TGA AGC	
**C. upsal-lpxA-R	TTC TAG CCC CTT GCT TGA TG	
***IAC-F	CTA ACC TTC GTG ATG AGC AAT CG	Deer et al., 2010
***IAC- R	GAT CAG CTA CGT GAG GTC CTA C	
***IAC-P	AGC TAG TCG ATG CAC TCC AGT CCT CCT	
27F	AGA GTT TGA TCM TGG CTC AG	Weisburg et al., 1991
1492R	TAC GGY TAC CTT GTT ACG ACT T	

*Primers used in qRT- PCR to detect *Campylobacter* genus, **Primers for multiplex PCR to detect *Campylobacter* species, ***Primer used as Internal Control template (IAC).

### Enrichment Conditions for Post Grit Samples from Wastewater Treatment Facilities

Raw sewage samples (post grit influent; PG) were collected from two different wastewater treatment facilities (Pine Creek and Bonnybrook) in Calgary, Alberta, in November and December, 2014. The samples were stored at 4–8°C and processed within 12 h after arrival. The wastewater samples (100 ml) were concentrated by centrifugation at 9000 rpm for 20 min at 4°C (Sorvall RC-5B), and pellets were resuspended in 4 ml of Bolton broth (Oxoid) for further enrichment process as described by Chenu et al. (2013) with minor modifications. Briefly, four different kinds of Bolton Broth (Oxoid) enrichment broth were prepared: (1) Bolton’s with *Campylobacter*-selective supplements [BN; cefoperazone 20 μg/ml, vancomycin 20 μg/ml, trimethoprim 20 μg/ml, and cycloheximide 50 μg/ml, Dalynn], (2) BN plus 10 μg/ml rifampicin [BNR], (3) BN plus 5 IU/ml polymyxin B [BNP], and (4) BN with both rifampicin and polymyxin B [BNRP]. Independently, 1 ml of pellet suspension was transferred to three wells in a 96-well plate and serially diluted to determine most probable number (MPN). For the 1st enrichment procedure, the plates were incubated at 37 or 42°C for 40–48 h under microaerobic conditions. Then, the culture broths were transferred to a 2nd enrichment medium consisting of the same antimicrobial supplements with 150 μg/ml 2,3,5-triphenyl-tetrazolium chloride (TTC, Sigma) and incubated for 24 h. TTC is a color indicator to show metabolic activity, and the inclusion of the dye in the assay aids in detection of levels of growth (Gabrielson et al., 2002). The cultures were subject to qRT-PCR and multiplex PCR.

### Validation of *C. jejuni* Growth with Antibiotic Supplements

*C. jejuni* ATCC 33560, which is a quality control (QC) strain for antimicrobial susceptibility testing of *C. jejuni* (Clinical and Laboratory Standards Institute [CLSI], 2010), and NCTC 11168 were employed to evaluate the growth capability of *C. jejuni* under different enrichment conditions. Four different kinds of enrichment broth were prepared as described above. *C. jejuni* ATCC 33560 and NCTC 11168 were cultured on MH agar plates at 42°C for 24 h and harvested with fresh MH broth. The bacterial suspension was adjusted to an OD_600_ of 0.07 and incubated at 42°C with shaking at 200 rpm under microaerobic conditions. To determine the growth of *C. jejuni* strains, the samples were taken at 0, 3, 6, 12, and 24 h, and CFU and OD_600_ values were measured.

### Confirmation of *Campylobacter* Growth using qRT-PCR

To confirm if *Campylobacter* was successfully enriched, 50 μl of culture broth was transferred to 96 well PCR plates and heated to 95°C for 10 min to extract DNA. Quantitative PCR was performed using an ABI 7500 (Applied Biosystems) system with *Campylobacter* genus-specific 16S rDNA primers (de Boer et al., 2013). The internal control template (IAC) and primers were included in reaction mixtures to measure inhibitory effects in enrichment samples (Deer et al., 2010). Amplification was carried out with following conditions: 50°C for 2 min and 95°C for 30 s; 40 cycles at 95°C for 3 s and 60°C for 30 s. *C*_t_ values were evaluated to determine the growth of *Campylobacter* and 3-tube MPN estimates.

### Confirmation of *Campylobacter* spp. using Multiplex PCR

To identify *Campylobacter* spp. in the enrichment broths, multiplex PCR was performed for 42°C enrichment broths as described elsewhere with primer sets for 16S rDNA and six species-specific primers (Yamazaki-Matsune et al., 2007). Same templates used in qRT-PCR were also employed for multiplex PCR. The amplification reaction was performed following conditions: 95°C for 15 min; 40 cycles at 95°C for 30 s, 58°C for 1 min and 30 s, 72°C for 1 min; 72°C for 7 min.

### Isolation of *Campylobacter* spp. and Identification of Non-*Campylobacter* Isolates by 16S rRNA Sequencing from Wastewater

To isolate *Campylobacter* spp. from enrichment cultures, wells showing the lowest *C*_t_ value in qRT-PCR results in the 2nd enrichment plate were selected. The cultures were prepared with 10-fold serial dilutions and sub-cultured on Bolton agar plates with Bolton supplement (BB, Dalynn) or Bolton agar plates with Preston supplement (BP, Oxoid). Following 2–3 days incubation at 42°C under microaerobic conditions, several colonies showing different shape, color, and transparency were randomly picked and transferred to the same fresh broth. After 2 days incubation, 50 μl of the cultures was harvested and boiled at 95°C for 10 min. Genus-specific 16S rDNA PCR amplification was carried out to distinguish between *Campylobacter* and non-*Campylobacter* (Linton et al., 1996). Amplification was performed following conditions: 94°C for 1 min; 35 cycles at 94°C for 30 s, 52°C for 30 s, 72°C for 1 min; 72°C for 5 min. PCR amplicons were visualized using 2% agarose gel with SYBR safe DNA gel stain solution (Invitrogen). To identify non-*Campylobacter* competitors growing in the selective enrichment conditions, 16S rDNA was amplified with universal bacterial domain primers (27F and 1492R) for 100 *Campylobacter* genus-specific 16S rDNA negative isolates (Weisburg et al., 1991). Amplification was conducted following conditions: 94°C for 1 min; 35 cycles at 94°C for 30 s, 50°C for 30 s, 72 °C for 1 min and 30 s; 72°C for 5 min. The amplified PCR products were purified and commercially sequenced by Sanger sequencing method (Macrogen, Inc., South Korea), and the results were analyzed by using Blastn^1^.

## Results

### *Campylobacter jejuni* Growth in the Presence of Additional Antibiotic Supplements

**FIGURE 1:**
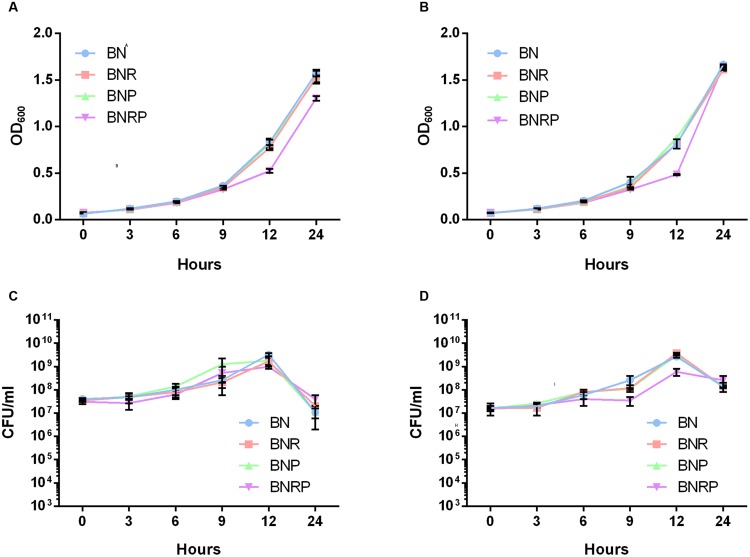
**Growth of *Campylobacter jejuni* ATCC 33560 and NCTC 11168 in four different antimicrobial enrichment conditions at 42°C.** Measurement of OD_600_ and CFU counting in *C. jejuni* ATCC 33560 **(A,C)** and *C. jejuni* NCTC 11168 **(B,D)**. **BN**, Bolton broth with Bolton *Campylobacter*-selective supplement; **BNR**, BN supplemented with rifampicin; **BNP**, BN supplemented with polymyxin B; **BNRP**, BN supplemented with and rifampicin and polymyxin B.

To improve the frequency of *C. jejuni* isolation from wastewater samples that are heavily contaminated with various microorganisms, we decided to increase antibiotic selective pressure by using different combinations of multiple antibiotics to which *C. jejuni* is intrinsically resistant (Taylor and Courvalin, 1988; Corry et al., 1995). For the growth testing, we used *C. jejuni* ATCC 33560, a QC strain for antibiotic susceptibility testing (Clinical and Laboratory Standards Institute [CLSI], 2010), and *C. jejuni* NCTC 11168, the first genome-sequenced *Campylobacter* strain (Parkhill et al., 2000). Whereas the Bolton selective supplement (BN) consists of three antibiotics, including cefoperazone, vancomycin, and trimethoprim, the Preston *Campylobacter*-selective supplement contains polymyxin B, rifampicin, and trimethoprim. The two selective supplements for *Campylobacter* isolation commonly contain trimethoprim. In the experiment, BN was used as basic antimicrobial supplements, and polymyxin B and/or rifampicin were added to BN to increase antibiotic selective pressure. The addition of either polymyxin B or rifampicin to BN did not affect the growth. The supplementation with both rifampicin and polymyxin B slightly reduced the OD_600_ at 12 h; however, there was no significant difference in growth in the four different enrichment conditions (**Figure 1**). The results indicate that *C. jejuni* can grow in the presence of combinations of the multiple antibiotics to which *C. jejuni* is naturally resistant.

### *C*_t_ Values of qRT-PCR in *Campylobacter* Detection under different Enrichment Conditions

**FIGURE 2:**
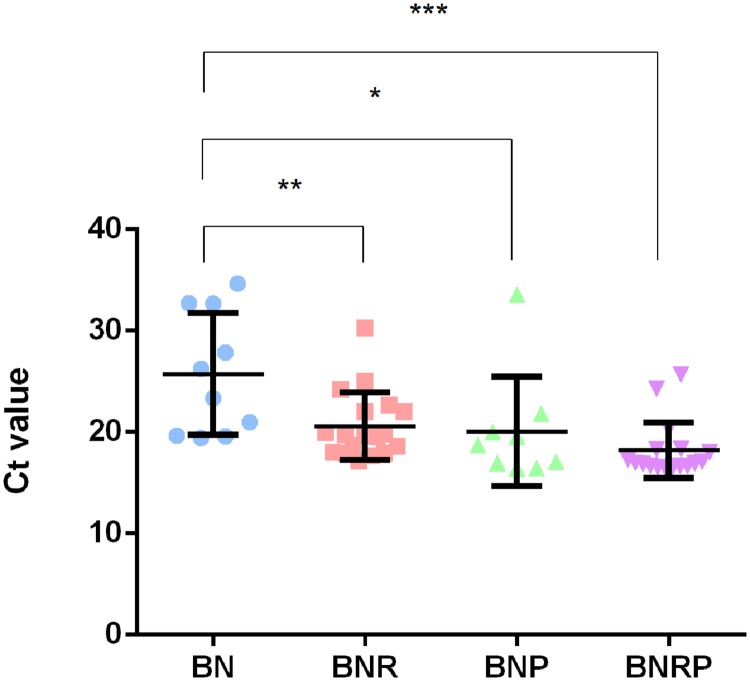
**Distribution of *C*_t_ values in four different enrichment conditions at 42°C.** Bolton broth with Bolton supplement (BN), BN with rifampicin (BNR), BN with polymyxin B (BNP), and BN with rifampicin and polymyxin B (BNRP). Statistical analysis was conducted with one-way analysis of variance (ANOVA) using GraphPad Prism 6 (GraphPad Software Inc., USA). *P ≤ 0.05, **P ≤ 0.01, and ***P ≤ 0.001.

The *C*_t_ values of qRT-PCR for the detection of *Campylobacter* varied depending on the antimicrobial enrichment. The addition of one of the antibiotics (i.e., either rifampicin or polymyxin B) significantly decreased the *C*_t_ value, meaning that *Campylobacter* population was increased by the selective enrichment. Furthermore, supplementation of both antibiotics showed the lowest *C*_t_ value compared to the other enrichment conditions (**Figure 2**), indicating that the increased antibiotic selective pressure enhanced the enrichment of *Campylobacter* in raw sewage samples. Positive samples were more frequently detected at 42°C than 37°C, and non-interpretable results, where *C*_t_ values could not be determined, were sometimes observed at 37°C (data not shown). This suggests that contaminating bacteria cannot be effectively inhibited at 37°C.

### Multiplex PCR Detection of *Campylobacter* spp. under different Enrichment Conditions

In addition to qRT-PCR detection, multiplex PCR was performed to determine the species of *Campylobacter* isolates. The results of multiplex PCR demonstrated that the primary *Campylobacter* spp. were *C. jejuni* and *C. coli* (**Table 2**). *C. jejuni* and *C. coli* were more frequently detected by the addition of rifampicin compared to polymyxin B. In many cases, positive results were discrepant between qRT-PCR and multiplex PCR (54% in qRT-PCR in comparison with multiplex PCR). For example, the same sample that was *Campylobacter*-negative based on qRT-PCR was shown to be positive by multiplex PCR (data not shown).

**Table 2:** The number of positive detection of *Campylobacter* 16S rDNA, *C. jejuni*, *C. coli*, and *C. lari* with multiplex PCR in four different enrichment conditions at 42°C.

Detection with multiplex PCR	BN	BNR	BNP	BNRP
16S rDNA only*	10 (40%)	3 (12%)	9 (33%)	2 (9%)
16S rDNA + *C. jejun*i	7 (28%)	9 (36%)	11 (41%)	9 (39%)
16S rDNA + *C. coli*	4 (16%)	6 (24%)	4 (15%)	7 (30%)
16S rDNA + *C. jejuni* + *C. coli*	3 (12%)	7 (28%)	2 (7%)	5 (22%)
16S rDNA + *C. lari*	1 (4%)	0	1 (4%)	0
Total 16s rDNA* positive	25 (100%)	25 (100%)	27 (100%)	23 (100%)

*Positive in multiplex PCR detection with *Campylobacter* 16S rDNA primers but negative for species-specific detection.

### Enhanced *Campylobacter* Isolation from Raw Sewage by Increased Antibiotic Selective Pressure

**FIGURE 3:**
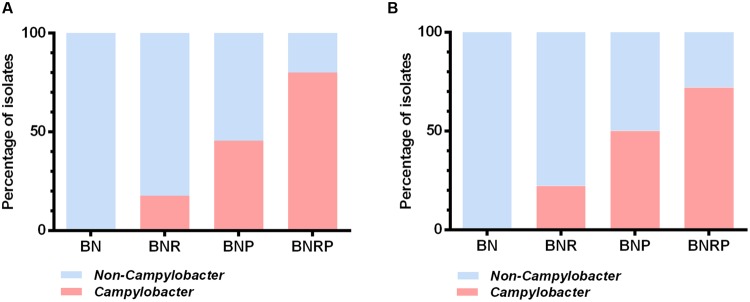
**Percentage distribution of *Campylobacter* and non-*Campylobacter* isolates in four different enrichment conditions at 42°C.** After antimicrobial enrichment, strains were isolated by growing on Bolton agar plates supplemented with Bolton selective supplement (BB; **A**) and Bolton agar plates supplemented with Preston selective supplement (BP; **B**). The results are based on PCR detection with primers for *Campylobacter* 16S rDNA. The number of isolates in BB is as follows; BN 18, BNR 17, BNP 22, and BNRP 25. The number of isolates in BP is as follows; BN 18 BNR 18, BNP 24, and BNRP 25.

The frequency of *Campylobacter* isolation from raw sewage was determined under the four different antibiotic enrichment conditions. To examine the effect of agar media on the *Campylobacter* isolation, we plated the enrichment cultures on Bolton and Preston agars, common culture media for *Campylobacter*. Consistent with the qRT-PCR results, the addition of rifampicin, polymyxin B, and both antibiotics significantly increased the isolation frequency for *Campylobacter* and decreased the isolation frequency of non-*Campylobacter* (**Figure 3**). In particular, BNRP showed the highest isolation rate of *Campylobacter*, whereas BN did not recover any *Campylobacter* spp. (**Figure 3**). Whereas the antibiotic enrichment significantly affected the isolation frequency, Bolton and Preston agar media did not make any differences in the isolation frequency (**Figure 3**). Morphologically, small pinkish or transparent colonies usually turned out to be *Campylobacter* (data not shown). To identify the major non-*Campylobacter* populations growing on the selective enrichment media, we randomly selected 100 colonies based on colony morphologies and performed 16S rDNA amplicon sequencing. The major non-*Campylobacter* spp. included *Enterococcus*, *E. coli*, *Klebsiella*, *Proteus*, and *Pseudomonas* (**Table 3**). The supplementation of additional antibiotics, either single (i.e., BNR and BNP) or both (i.e., BNPR), suppressed the growth of other bacterial populations. However, *Enterococcus* spp., such as *Enterococcus durans* and *Enterococcus faecium*, were still isolated in BNPR (**Table 3**). Importantly, increased antibiotic selective pressure improved the frequencies of isolating *Campylobacter* from wastewater (**Table 3**).

**Table 3:** Distribution of *Campylobacter* and non-*Campylobacter* strains in four different enrichment conditions at 42°C.

Species	BN	BNR	BNP	BNRP
*E. coli*	9 (25%)	10 (28.6%)	1 (2.2%)	0
*E. fergusonii*	3 (8.3%)	4 (11.4%)	0	0
*E. durans*	0	5 (14.3%)	7 (15.2%)	8 (16%)
*E. faecium*	4 (11.1%)	6 (17.1%)	6 (13%)	4 (8%)
*P. aeruginosa*	5 (13.9%)	3 (8.6%)	5 (10.9%)	0
*P. penneri*	0	0	1 (2.2%)	0
*P. mirabilis*	0	0	4 (8.7%)	0
*K. pneumoniae*	15 (41.7%)	0	0	0
*Campylobacter*	0	7 (20%)	22 (47.8%)	38 (76%)
Total number of isolates	36 (100%)	35 (100%)	46 (100%)	50 (100%)

## Discussion

In this study, we improved the efficacy of *C. jejuni* isolation from wastewater by increasing antibiotic selective pressure in the enrichment step. The addition of rifampicin, polymyxin B, or both to the enrichment media affected the *C*_t_ values of qRT-PCR results (**Figure 2**). According to the distribution of *C*_t_ values, the addition of the antibiotic(s) decreased *C*_t_ values, meaning that antibiotic supplements improved the growth of *Campylobacter*. In particular, rifampicin significantly reduced *C*_t_ values (**Figure 2**). A few studies have thus far reported that increased selective pressure enhances *Campylobacter* isolation from food. Yoo et al. (2014) reported that the addition of rifampicin (10 μg/ml) or polymyxin B (5 IU/ml) to Bolton agar (Bolton agar with Bolton supplement) restrained the growth of non-*Campylobacter* without any inhibition of *C. jejuni* and *C. coli* in fresh produce foods. Chon et al. (2013) demonstrated that the addition of high concentrations of polymyxin B to the mBolton supplement in enrichment procedure improved the efficiency of *C. jejuni* and *C. coli* recovery and suppressed background competing bacteria. Consistently, our results showed that the supplementation with additional antibiotics improved the efficacy of *C. jejuni* isolation even from heavily contaminated wastewater samples. In addition, we also identified bacterial populations that compete with *Campylobacter* under the four different selective enrichment conditions. The inputs of *Campylobacter* entering the influent of wastewater treatment facilities in this study would be primarily from sewage effluent in Calgary and also possibly from wildlife, such as migrating birds (Cody et al., 2015). Depending on the treatment procedure, the incidence rate of *Campylobacter* in sewage effluent can be altered, and cross contamination between water resources and sewage is associated with water-borne *Campylobacter* outbreaks (Jones, 2001; Pitkanen, 2013).

The 16S rDNA amplicon sequencing analysis of individual colonies from the enrichment plates revealed that *Escherichia*, *Pseudomonas*, *Klebsiella*, and *Enterococcus* were the major competing bacteria in *C. jejuni* isolation from wastewater (**Table 3**). Baylis et al. (2000) identified competitor organisms in foods by using Preston and Bolton selective supplement media, showing that *Yersinia, Enterobacter*, *Escherichia*, *Enterococcus*, *Pseudomonas*, and *Klebsiella* are representative competitors. This is quite similar to our results from the BN enrichment conditions. *Escherichia* were frequently isolated in BN (**Table 3**), presumably because extended-spectrum beta-lactamase (ESBL)-producing *E. coli* may reduce the selectivity of Bolton supplement and consequently *E. coli* growth would suppress *Campylobacter* (Moran et al., 2011). Although the supplementation of additional antibiotic(s) suppressed the overgrowth of competing bacteria and enriched *Campylobacter*, *Enterococcus* survived well in the presence of five different antibiotics as it was frequently isolated with *Campylobacter* (**Table 3**). The survival of *Enterococcus* in the presence of vancomycin (20 μg/mL) in Bolton supplement indicated that *Enterococcus* isolated from the enrichment broth is vancomycin-resistant enterococci (VRE), a drug-resistant strain of serious public health concern (Cetinkaya et al., 2000). This study aimed at developing an improved culture method to isolate *Campylobacter* from wastewater, and we used the influent samples, not the effluent, since the influent is more contaminated than the effluent. Therefore, the results do not provide the information about the level of *Campylobacter* contamination in the effluent that may have a direct impact on public health compared to the influent data.

In this study, we demonstrated that antibiotic selective pressure and culture temperature are the critical factors for *C. jejuni* isolation from raw sewage. The BN, BNR, BNP, and BNRP conditions showed similar MPN values at 42°C; however, only BNRP showed reasonable MPN values and BNR and BNP showed relatively lower MPN numbers at 37°C compared to those at 42°C (data not shown). The results exhibited that culture temperature also plays an important role in the selective enrichment of *C. jejuni*. Humphrey et al. showed the effect of antibiotics and temperature on the recovery rate in cold-damaged *C. jejuni*. The sub-lethally injured cells are more sensitive to antibiotics in 43°C than 37°C, affecting the restoration of *C. jejuni* (Humphrey, 1986). In previous studies, Humphrey et al. also suggested that pre-incubation at 37°C for 4–18 h followed 42 or 37°C incubation for 48 h would be beneficial to the recovery of *Campylobacter* in comparison with 42°C (Humphrey, 1989; Humphrey and Muscat, 1989). Whereas Khan et al. (2013) demonstrated that the detection frequency of *Campylobacter* spp. was higher at 37°C in BN than 42°C, *C. jejuni* was detected more frequently at 42°C than at 37°C. Consistently, our results suggested that 42°C seems to enhance *C. jejuni* growth in raw sewage samples.

The additional antibiotic(s) plus an increased incubation temperature (i.e., 42°C) improved the isolation rates of *C. jejuni* and *C. coli* from heavily contaminated raw sewage samples. The addition of rifampicin and polymyxin B specifies the selective enrichment of thermo-tolerant *Campylobacter* spp., such as *C. jejuni* and *C. coli*, the major human pathogenic species (Kaakoush et al., 2015). Based on our findings, increased antibiotic selective pressure and culture temperature are the key parameters impacting the success in *C. jejuni* isolation from heavily contaminated wastewater samples. Additionally, rifampicin appears to be effective in improving the selectivity of *Campylobacter* enrichment for PCR-based quantitative methods, whereas both rifampicin and polymyxin B are required to suppress competing bacterial growth and improve the selectivity of *C. jejuni* isolation with culture-based methods.

## Author Contributions

Design of the project: JK, NA, NN, and BJ; Performance of the experiments: JK, EO, GB, and SB; Data analysis: JK, EO, GB, SB, LC, NA, NN, and BJ; Writing of the manuscript: JK, NA, and BJ.

## Conflict of Interest Statement

The authors declare that the research was conducted in the absence of any commercial or financial relationships that could be construed as a potential conflict of interest.

## Footnotes

1 http://blast.ncbi.nlm.nih.gov/Blast.cgi
